# The ectodysplasin-A receptor is a candidate gene for lateral plate number variation in stickleback fish

**DOI:** 10.1093/g3journal/jkac077

**Published:** 2022-04-04

**Authors:** Telma G Laurentino, Nicolas Boileau, Fabrizia Ronco, Daniel Berner

**Affiliations:** 1 Department of Environmental Sciences, Zoology, University of Basel, 4051 Basel, Switzerland; 2 Department of Environmental Science, Policy, and Management, University of California, Berkeley, CA 94720, USA

**Keywords:** *Gasterosteus aculeatus*, genetic architecture, genome scan, lateral plates, pooled sequencing, population genomics

## Abstract

Variation in lateral plating in stickleback fish represents a classical example of rapid and parallel adaptation in morphology. The underlying genetic architecture involves polymorphism at the ectodysplasin-A gene (*EDA*). However, lateral plate number is influenced by additional loci that remain poorly characterized. Here, we search for such loci by performing genome-wide differentiation mapping based on pooled whole-genome sequence data from a European stickleback population variable in the extent of lateral plating, while tightly controlling for the phenotypic effect of *EDA*. This suggests a new candidate locus, the *EDA* receptor gene (*EDAR*), for which additional support is obtained by individual-level targeted Sanger sequencing and by comparing allele frequencies among natural populations. Overall, our study illustrates the power of pooled whole-genome sequencing for searching phenotypically relevant loci and opens opportunities for exploring the population genetics and ecological significance of a new candidate locus for stickleback armor evolution.

Significance Much remains to be learned about the genetic basis of phenotypic variation among natural populations. Performing genome scans in threespine stickleback fish, we search for genetic loci contributing to variation in lateral plating and identify a strong novel candidate gene, the *EDA* receptor *EDAR*.

## Introduction

Adaptive diversification among populations is ubiquitous (Mousseau *et al.* 2000; Schluter 2000; Leimu and Fischer 2008; Hereford 2009), but much remains to be learned about its genomic basis. The latter is important because information on the genetic architecture of adaptation helps understand how selection shapes genome-wide genetic variation within and among populations (Flaxman *et al.* 2014; Yeaman 2015; Berner and Roesti 2017; Villoutreix *et al.* 2021), to what extent genetic variation is used repeatedly for adaptation in independent populations (parallel evolution; Arendt and Reznick 2008; Ralph and Coop 2010; Martin and Orgogozo 2013; Thompson *et al.* 2019), or where adaptive genetic variation originates and how it is maintained (Barrett and Schluter 2008; Messer and Petrov 2013; Galloway *et al.* 2020; Haenel *et al.* 2022). Information on the genetic architecture of adaptive diversification further provides a crucial resource for elucidating the developmental basis of evolution.

**Fig. 1.**
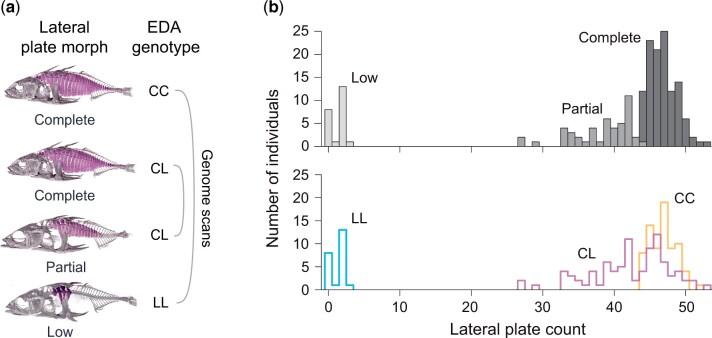
Experimental groups and distribution of lateral plate number in the natural population. a) Computed tomography scans of a representative specimen from each of the three lateral plate morphs, with the plates colored purple. The experimental groups underlying the genome scans combined phenotypic lateral plate morph with the genotype at the *EDA* locus. The two focal group comparisons are indicated by round brackets. b) Distribution of total lateral plate count (all plates beyond the pelvic girdle on both body sides) among 186 stickleback from the natural population (upper panel). The plate morphs are separated by different gray shades (the bars are stacked, no overlap). The lower panel shows separate histograms for the three *EDA* genotype classes, revealing the broad range of plate counts in *EDA* heterozygotes (CL).

An organismal system in which progress in uncovering the genetic architecture of phenotypic diversification has been made is the threespine stickleback (*Gasterosteus aculeatus*) (e.g. Miller *et al.* 2007; Chan *et al.* 2010; Howes *et al.* 2017; Cleves *et al.* 2018), a fish exhibiting extensive population diversification when adapting from its ancestral marine habitat to novel freshwater habitats (Bell and Foster 1994). One classical trait evolving rapidly and repeatedly in stickleback upon freshwater colonization is the number of lateral plates (Bell *et al.* 2004; Kristjánsson 2005; Le Rouzic *et al.* 2011; Lescak *et al.* 2015), which represent a component of the fish’s bony armor protecting against predators (Reimchen 1992, 2000; Leinonen *et al.* 2011). While pelagic (i.e. open water) populations in marine environments are generally completely plated, with their flanks covered from the head to the tail fin by lateral plates (hereafter “Complete morph”), freshwater stickleback typically lack the plates posterior to the pelvic girdle altogether (“Low morph”), or at least partially (“Partial morph”) (Fig. 1a). This plate reduction has evolved numerous times independently by parallel selection of standing genetic variation at ectodysplasin-A (*EDA*) (Colosimo *et al.* 2004, 2005; Cresko *et al.* 2004; Jones *et al.* 2012; Berner *et al.* 2014; Roesti *et al.* 2014, 2015; Terekhanova *et al.* 2014; Lescak *et al.* 2015; Nelson and Cresko 2018), a gene widely implicated in the development of vertebrate ectodermal tissues such as teeth (Mikkola and Thesleff 2003; Cui and Schlessinger 2006; Wucherpfennig *et al.* 2019) and scales (Harris *et al.* 2008; Iida *et al.* 2014). In laboratory crosses between completely and low plated stickleback, allelic polymorphism at the *EDA* locus explains approximately 75% of the phenotypic variation (Colosimo *et al.* 2004; Cresko *et al.* 2004; Berner *et al.* 2014). Lateral plate evolution in stickleback is thus often strongly driven by *EDA*. Nevertheless, the presence of other factors influencing variation in lateral plating in natural populations has been suggested (Colosimo *et al.* 2004; Knecht *et al.* 2007; Lucek *et al.* 2012a; Indjeian *et al.* 2016; Yamasaki *et al.* 2019).

The objective of the present study is to search for genetic factors beyond *EDA* influencing lateral plate variation in a natural population of threespine stickleback. We focus on fish from the Lake Constance basin in Central Europe, a system including a large lake population adapted to a pelagic life style, and several neighboring populations residing in (generally small) tributary streams and exhibiting a benthic life style (Berner *et al.* 2010; Lucek *et al.* 2012b; Moser *et al.* 2012; Roesti *et al.* 2015; Marques *et al.* 2016). The lake fish are almost consistently completely plated like marine fish, whereas the stream populations generally tend toward reduced plating, thus showing substantial proportions of partial and low morphs (Moser *et al.* 2012; Roesti *et al.* 2015). Marker-based signatures at the *EDA* locus indicate that selection favors extensive plating in the pelagic lake population presumably highly exposed to predators (Roesti *et al.* 2015). In contrast, shelter from predators likely renders plating costly in the benthic stream populations (Reimchen 1992; Bergstrom 2002; Leinonen *et al.* 2011).

In this study, we take advantage of the variation in lateral plating in one of these stream populations and the power of pooled whole-genome sequencing to search for loci contributing to lateral plate variation while controlling for the effect of *EDA*. The evidence of a novel candidate locus discovered in this way is then strengthened by targeted Sanger sequencing and the comparison of allele frequencies among multiple natural populations from different environments.

## Materials and methods

### Study population, lateral plate phenotyping, and *EDA* genotyping

Our study focuses on a stream population in which the partial plate morph occurs at a relatively high frequency [the NID population in Berner *et al.* (2010), also referred to as “COW stream” in Moser *et al.* (2012)]. To characterize variation in lateral plating within this population, we phenotyped 297 adult individuals (102 males, 195 females) captured for a different experiment (Berner *et al.* 2017). All lateral plates posterior to the pelvic girdle (including the plates forming the caudal keel) were counted by the same person (TGL) under a dissecting microscope on both sides of the fish, and every gap in plating, and its position, was recorded. Based on this information, a subset of 186 individuals was assigned to one of three different lateral plate morphs for subsequent genomic analysis (Fig. 1a): low plated individuals exhibited no more than three plates posterior to the pelvic girdle and no keel plates on the caudal peduncle; partially plated individuals exhibited a continuous gap of at least three plates in the mid-body region (typically located between plates 11 and 21) on both sides of their body, and a keel on the caudal peduncle; completely plated individuals displayed a continuous series of plates from the pelvic girdle to the tip of the caudal peduncle on both sides of their body, thus also including a keel. The remaining 111 individuals among the total 297 phenotyped individuals exhibited minor and sometimes asymmetric plate reduction relative to the complete morph; to obtain clear-cut phenotypic categories for pooled sequencing and genetic mapping, these individuals were ignored.

Fin tissue samples from the 186 individuals assigned to plate morphs were next subjected to genomic DNA extraction with the Zymo Quick-DNA Miniprep Plus kit. We followed the manufacturer’s protocol, with the modification that the lysate resulting from protease digest was centrifuged, and DNA was extracted from the supernatant only. We also included an RNAse treatment (4 μl, 100 mg/ml, for 5 min). Because our aim was to discover loci other than *EDA* that potentially influence lateral plating, our differentiation mapping approach required precise knowledge of *EDA* genotypes. Each individual was therefore genotyped for an indel (insertion–deletion) polymorphism within intron 1 of *EDA* amplified by the marker Stn382 (Colosimo *et al.* 2005). The two fragment length alleles at this polymorphism are generally assumed to cosegregate reliably with the two *EDA* alleles (i.e. complete and low), allowing us to classify each individual as homozygote for the complete (CC) or low (LL) allele, or as heterozygote (CL). Throughout our paper, we indicate *EDA* genotypes by superscripts.

### Pooled whole-genome sequencing, alignment, and nucleotide pileup

Combining lateral plate morph with *EDA* genotype, each individual was assigned to one of four categories for subsequent pooled whole-genome sequencing (poolSeq) (Fig. 1a): Complete^CC^ (*n *=* *74); Low^LL^ (*n *=* *23); Complete^CL^ (*n *=* *42); and Partial^CL^ (*n *=* *47). The latter two categories represent stickleback with the same genotype at *EDA*, but exhibiting distinct lateral plate morphs. After measuring individual DNA concentrations with a Qubit fluorometer using the Broad Range kit (Invitrogen, Thermo Fisher Scientific, Wilmington, DE, USA), DNA from all individuals within each of the four categories was combined in equimolar proportion into a single library. The four resulting DNA libraries were then barcoded individually and paired-end sequenced without PCR amplification to 151 base pairs on an Illumina HiSeq2500 instrument. Each library was sequenced on two lanes, yielding a median read depth per base of 65× (Complete^CC^), 71× (Low^LL^), 118× (Complete^CL^), and 100× (Partial^CL^). This combination of read depth and number of individuals is expected to allow estimating allele frequencies within groups with relatively high precision (Ferretti *et al.* 2013; Gautier *et al.* 2013; Berner 2019).

Raw sequence data were parsed by experimental group according to barcode, and aligned to the third-generation assembly of the threespine stickleback reference genome (Glazer *et al.* 2015) with Novoalign 3.03.00 (http://www.novocraft.com/products/novoalign) (options: -F STDFQ -t 540 -g 40 -x 12 -r N -e 200 -i PE 200,250). Using the Rsamtools R package (Morgan *et al.* 2017), the alignments were converted to BAM format, and nucleotide counts were performed for every genomic position by using the pileup function.

### Genome-wide differentiation mapping

Our main approach to searching for loci beyond *EDA* influencing lateral plating was a genomic comparison between the Complete^CL^ and the Partial^CL^ groups (Fig. 1a). The underlying rationale was that if additional loci with a substantial influence on plating occur in our study population, they should exhibit exceptionally strong allele frequency differentiation between these two groups differing in plate phenotype while being genotypically identical at the *EDA* locus.

In an initial step, however, we performed a genomic comparison of the Complete^CC^ vs Low^LL^ groups to confirm the reliability of our *EDA* genotyping. For this, we determined the magnitude of genetic differentiation between these two groups across all genome-wide SNPs (throughout our study, genetic differentiation is quantified by the absolute allele frequency difference *AFD*; Berner 2019). The SNPs for this analysis were required to exhibit a read depth between 40× and 130× within each group to exclude poorly sequenced and repeated regions (details provided in Supplementary Fig. 1). Moreover, a minor allele frequency of at least 0.2 across the two groups pooled was required to exclude sequencing errors (the Illumina HiSeq2500 instrument has a sequencing error rate <0.003; Stoler and Nekrutenko 2021) and to ensure adequate information content (Roesti *et al.* 2012). This strategy yielded 1,127,066 SNPs across the 447 megabase (Mb) stickleback genome. In addition to evaluating differentiation at the individual SNPs, we smoothed the data by averaging *AFD* across sliding windows of 40 kb width with 20 kb overlap, requiring a minimum of six SNPs per window. Averaging with a higher resolution (20- or 10-kb windows) produced similar results supporting the same conclusions.

For the actual Complete^CL^ vs Partial^CL^ comparison, we proceeded analogously, except that SNPs were here required to exhibit a read depth between 40× and 200× within each group (Supplementary Fig. 1), yielding 1,247,920 total markers. As a robustness check, the Complete^CL^ vs Partial^CL^ comparison was repeated as described, except that the sequence reads were aligned to an independent, scaffold-level genome assembly (Berner *et al.* 2019) derived from an individual from the same population (NID, Lake Constance basin) from which the experimental individuals were sampled. We here raised the minimum read depth threshold to 60× within each group to increase analytical stringency, thus obtaining 1,052,453 total markers.

### Identification of candidate loci and gene annotation

For the group comparison Complete^CL^ vs Partial^CL^—the main focus of this paper, we defined candidate loci potentially influencing lateral plating by identifying the ten SNPs showing the highest between-group *AFD* values genome-wide (roughly corresponding to the top 0.001% of the *AFD* distribution). With this analytical stringency, we explicitly focused on loci with relatively large phenotypic effect only. Each of these loci was annotated by extracting from the reference genome annotation all genes located within a 180-kb window centered at the candidate SNP (or SNP cluster). For each resulting transcript ID, we retrieved gene name, gene ontology information, strand, and transcript start and end positions from the *ensemble* bioMART stickleback database (www.ensembl.org/biomart). Every gene was then evaluated for a role in bone or ectodermal development in humans and/or zebrafish by using the *gene cards* (www.genecards.org) and *Zfin* (www.zfin.org) databases. Genes were further subjected to literature search for whether they were connected to the tumor necrosis factor pathway (which includes *EDA*), or the *Wnt*/beta-catenin pathway (which interacts with the *EDA* pathway; O’Brown *et al.* 2015).

### Strengthening the evidence of a candidate locus by individual Sanger sequencing

The above candidate gene search based on differentiation mapping with poolSeq data suggested a role for a polymorphism near the *EDA* receptor (*EDAR*) in lateral plate variation. To strengthen the evidence for this candidate locus, we performed targeted Sanger sequencing around the SNP showing the highest *AFD* between the Complete^CL^ and Partial^CL^ groups at this locus. For this, we used a “validation panel” of 46 independent individuals collected for previous studies and not included in the poolSeq-based mapping.

The validation panel included individuals chosen to display the partially plated phenotype based on the same criteria as applied in our original screen, and hence to be heterozygous at the *EDA* locus (see below). These individuals originated from Lake Constance (*n *=* *15), from NID stream (*n *=* *12), or were F2 hybrids derived from these populations for the experiment reported in Laurentino *et al.* (2020) (*n *=* *19). We predicted that if the target polymorphism at the *EDAR* locus was associated with plate reduction, our validation panel should be enriched for the allele identified to be associated with reduced plating (hereafter the “partial allele”) relative to the expectation based on the natural population frequency. Combining individuals from the lake and stream with their F2 hybrids was adequate because the natural lake and stream populations were found to exhibit an almost identical frequency of the partial allele (lake 0.581; stream 0.587). DNA from the individuals of the validation panel was extracted as described above, and PCR was performed using the primers and conditions specified in Supplementary Analysis 1. PCR products were sequenced on an ABI3130xl instrument (Applied Biosystems) and genotyped in FinchTV (https://digitalworldbiology.com/FinchTV).

To evaluate the compatibility of the validation panel’s allele frequency with the random expectation, we first predicted Hardy–Weinberg proportions for all three diploid genotype classes by assuming a population frequency of the partial plating allele of 0.583 (i.e. the average of the natural lake and stream frequencies). Then we calculated the observed deviance from this expectation as the sum of the squared difference between the observed and predicted genotype frequencies across the three genotype classes. The magnitude of this statistic was then evaluated against a random distribution obtained by generating random panels of 46 diploid individuals 9,999 times according to the population allele frequency, and calculating the deviance for each of these iterations (this evaluation was two-tailed).

### Evidence from allele frequencies in natural populations

Beside evidence for our new candidate locus from differentiation mapping and known gene functions, we sought to obtain additional support from the tendency of specific alleles to be associated with specific ecological environments among populations. To investigate such allele–environment relationships, we inspected the frequency of both the *EDA* low allele and the partial allele at the new *EDAR* candidate locus in natural marine and freshwater populations. We predicted that the alleles reducing lateral plating should tend to be rare in the ancestral marine habitat where stickleback are selected for complete lateral plating, but display higher frequencies in freshwater—a pattern generally observed for *EDA* (e.g. Colosimo *et al.* 2005).

To examine this prediction, we complemented our data from the NID stream population by published pooled whole-genome sequence data from the ROM Lake Constance population located in the same watershed (Bissegger *et al.* 2020; Laurentino *et al.* 2020), from three additional freshwater samples [Misty Lake, Vancouver Island, Canada, Haenel *et al.* (2021); plus two samples from North Uist, Outer Hebrides, Scotland, Haenel *et al.* (2022)], and from six Atlantic marine stickleback samples (Germany, Ireland, Scotland, Iceland, Canada, and the Netherlands, Haenel *et al.* 2022). These pools combined DNA from 21 to 240 individuals and were sequenced to 66–260× read depth.

For each of these pools, we determined and plotted the frequency of the SNP alleles associated with reduced plating identified in the above genome scans. For *EDA*, we here considered all SNPs (*n *=* *409) proving fixed between the Complete^CC^ and Low^LL^ groups. For the *EDAR* candidate locus, we considered the top-*AFD* SNP from the Complete^CL^ vs Partial^CL^ comparison, and all flanking markers exhibiting differentiation of at least 0.35 (i.e. at least 5.5 times genome-wide median *AFD*; *n *=* *16 SNPs).

## Results and discussion

### Phenotypic variation in lateral plating and associated *EDA* genotypes

Our phenotypic analysis confirmed high variability in lateral plating in our focal stream stickleback population (Fig. 1b) (Berner *et al.* 2010; Moser *et al.* 2012; Roesti *et al.* 2015). The majority (116, 62%) of the 186 individuals that could be assigned unambiguously to a plate morph according to our criteria proved completely plated, 47 (25%) partially plated, and 23 (13%) low plated. Median total plate count for these morphs was 47, 39, and 2. We observed no individuals with less than ten plates but exhibiting a keel, a phenotype reported from Icelandic freshwater stickleback (Lucek *et al.* 2012a).

Genotyping the same 186 individuals at the *EDA* locus revealed that low plated fish were always homozygous for the low allele, and partially plated individuals were always heterozygous. Completely plated fish, in turn, were either heterozygous (45%), or homozygous for the *EDA* complete allele (55%). Combining the phenotypic data with *EDA* genotypes thus revealed that individuals heterozygous at the *EDA* locus covered a wide range of plate phenotypes, as expected if genetic factors beyond *EDA* influence lateral plating in the NID population.

### Genome-wide differentiation mapping

**Fig. 2.**
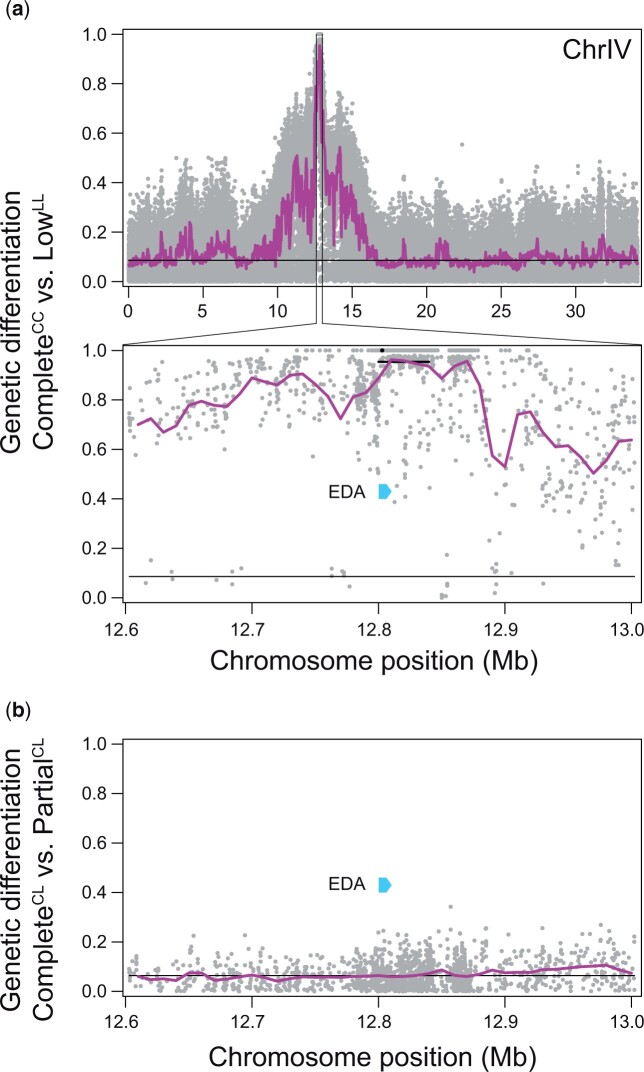
a) Genetic differentiation, quantified by the absolute allele frequency difference *AFD*, between the Complete^CC^ and Low^LL^ groups. The dots represent individual SNPs and the black horizontal lines indicate genome-wide median differentiation in this comparison. In the upper panel, differentiation is shown along the entire chromosome IV. The purple profile shows differentiation smoothed across 40 kb sliding windows with 20 kb overlap. The lower panel is a close-up into the 400 kb segment centered at the *EDA* locus. The purple profile here reflects smoothing using 20 kb sliding windows with 10 kb overlap. The black dot denotes a SNP in immediate proximity to the fragment length polymorphism used for *EDA* genotyping, and the black horizontal bar indicates the average differentiation across the 40 kb window exhibiting the greatest genome-wide differentiation between the groups. The location of the *EDA* gene is given as blue arrow. In (b), genetic differentiation is visualized analogously across the same 400 kb segment, but based on the Complete^CL^ vs Partial^CL^ genome scan.

To validate our strategy of searching for genomic regions involved in lateral plating based on poolSeq for combinations of plate morph by *EDA* genotype, we first mapped differentiation between the Complete^CC^ and the Low^LL^ groups across the stickleback genome. This genome scan identified the neighborhood of the *EDA* gene as the only strongly differentiated genome region, with hundreds of SNPs across ca. 200 kb showing complete differentiation in allele frequency (i.e. *AFD *=* *1) between the groups (median *AFD* across all genome-wide SNPs: 0.086) (Fig. 2a; differentiation profiles across all chromosomes are presented in Supplementary Fig. 2). This finding confirmed that our genotyping of individuals for *EDA* alleles of major phenotypic effect based on an indel within this gene was highly reliable.

Mapping genetic differentiation between the Complete^CL^ and Partial^CL^ categories in the same way identified SNPs reaching differentiation up to 0.541 (genome-wide median *AFD*: 0.064; differentiation profiles across all chromosomes are show in Supplementary Fig. 3). The ten most strongly differentiated SNPs genome-wide (*AFD* ≥ 0.486) were selected for the exploration of candidate genes. These SNPs included a single marker on the chromosomes II, III, XVI, and XVIII, a cluster of four markers on chromosome XX, and two SNPs on a scaffold unanchored to chromosomes. This genome scan also made clear that variation in plating between Complete^CL^ and Partial^CL^ stickleback is not influenced by additional genetic variation in the *EDA* region (Fig. 2b).

### *EDAR* is a candidate gene for variation in lateral plating

**Fig. 3.**
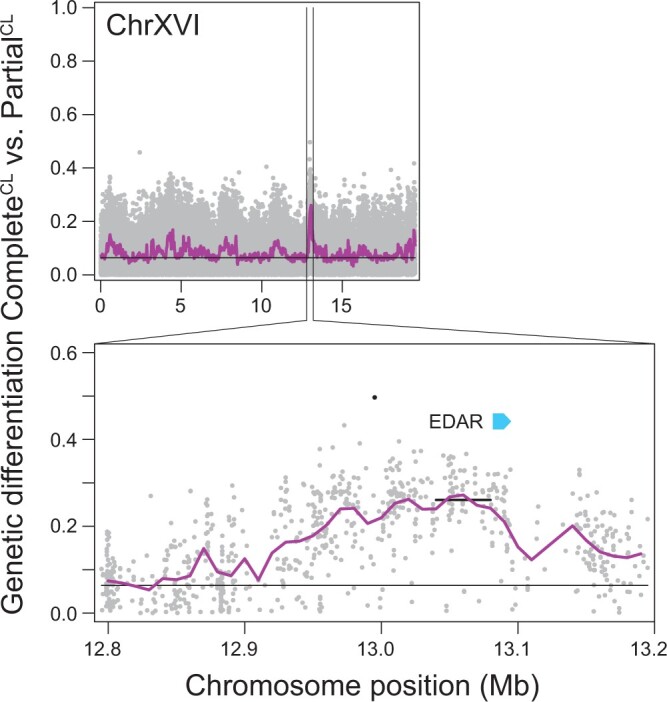
Genetic differentiation (*AFD*) in the Complete^CL^ vs Partial^CL^ group comparison, shown for the entire chromosome XVI (top), and for a 400-kb segment containing the *EDAR* gene (bottom). In the latter, the black dot represents one of the ten high-differentiation SNPs selected for candidate gene search, and the black horizontal bar gives the average differentiation across the 40 kb sliding window exhibiting the greatest genome-wide differentiation in this group comparison. All other graphing conventions follow Fig. 2.

Annotating the regions containing the ten most divergent SNPs in the Complete^CL^ vs Partial^CL^ genome scan yielded a highly suggestive candidate gene for lateral plate variation. Specifically, one of these markers, together with numerous flanking SNPs, formed a distinct peak of high differentiation on chromosome XVI (Fig. 3). The marker showing the strongest differentiation in this region (*AFD *=* *0.497) was located in a noncoding segment 86.5 kb upstream of the coding region of *EDAR*, the only annotated gene ontology for “bone development” within all chromosome segments screened for candidate genes. We hereafter refer to this region as the *EDAR* locus. Repeating our differentiation mapping based on an independent genome assembly derived from a specimen from the NID population confirmed the methodological robustness of the identification of the *EDAR* locus: in this alternative genome scan performed with higher statistical stringency, the SNP exhibiting the second highest differentiation value genome-wide (*AFD *= 0.478) was located on a scaffold segment homologous to the *EDAR* locus in the original genome scan, and coincided exactly with the original top-differentiation SNP at the *EDAR* locus (Supplementary Fig. 4). Irrespective of the genome assembly used for read alignment, the *EDAR* locus harbored the sliding window showing the strongest average differentiation between Complete^CL^ and Partial^CL^ stickleback genome-wide (Fig. 3; Supplementary Fig. 4).

*EDAR* is the cell-surface receptor to which the *EDA* protein binds for triggering ectodermal development (Knecht *et al.* 2007). This gene is widely implicated in the formation of fish ectodermal structures such as scales (Harris *et al.* 2008; Iida *et al.* 2014; Kondo *et al.* 2001) and other dermal bony tissues derived from scales (Cheng *et al.* 2015; Shono *et al.* 2019). Furthermore, polymorphism at *EDAR* was associated with subtle variation in lateral plate number (range: 2 plates) in an artificial cross in stickleback, albeit only in low plated individuals homozygous for the *EDA* low allele (corresponding to Low^LL^ fish in our study) (Knecht *et al.* 2007). Interestingly, after *EDA*, *EDAR* has the highest number of putative regulatory regions among all members of the *EDA* signaling pathway, thus potentially promoting the modulation of *EDA* signaling specific to developmental phases and tissues (Knecht *et al.* 2007). Collectively, this functional evidence supports *EDAR* as a strong candidate gene for lateral plate variation in our stickleback population.

Apart from the *EDAR* locus, our examination of the nine other high-differentiation SNPs produced no strong candidate gene. These SNPs either showed minimal read depth just passing our lower threshold so that their high *AFD* value likely represents sampling stochasticity (e.g. the four SNPs on ChrXX were not supported by the genome scan performed with higher stringency); lacked support in the form of elevated differentiation across *multiple* markers flanking the top-differentiation SNPs (Chrs II, III, XVIII, XX; Supplementary Figs. 3 and 5); and/or showed no genes relevant to our search criteria in their physical neighborhood (Chrs XVIII, XX; Supplementary Fig. 5). Nevertheless, we present the full gene annotations around the high-differentiation SNPs, and a discussion of the functional evidence for the subset of associated genes qualifying as potentially functionally relevant according to our criteria, in Supplementary Fig. 5. We also acknowledge that our analytical approach may miss additional weaker genotype–phenotype associations present in our data, or that such associations may have emerged if we had performed our genome scan with higher statistical precision (i.e. more individuals per group).

### Support for the *EDAR* candidate locus from Sanger sequencing

**Fig. 4.**
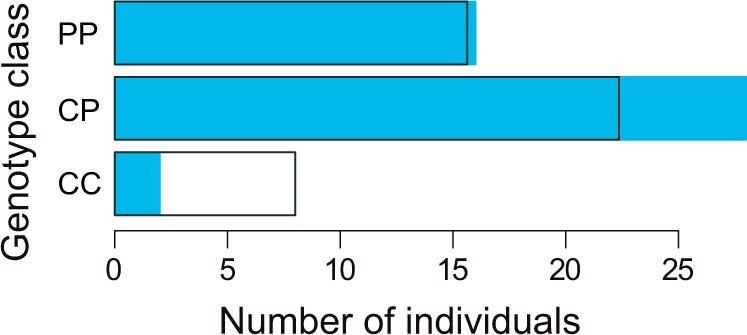
Exploring *EDAR* genotypes by targeted sequencing of the validation panel. Shown are counts of the three genotype classes (P = partial allele; C = complete allele) at the top-differentiation SNP upstream of the *EDAR* gene (black dot in Fig. 3 bottom) among 46 partially plated individuals not included in the genome scans. The blue bars show the empirically observed counts while the black rectangles indicate the counts expected from the natural population allele frequencies at this polymorphism.

All 46 partially plated individuals from the validation panel produced robust PCR products for the DNA segment covering the top-differentiation SNP at the *EDAR* locus. In agreement with our expectation, these individuals proved enriched for the *EDAR* partial allele (genotype data given in Supplementary Analysis 1). Specifically, we observed a deficit of individuals homozygous for the complete allele, and an excess of heterozygotes (Fig. 4). The observed genotype counts were relatively poorly compatible with random sampling from the natural populations (two-tailed *P *=* *0.09; the observed deviance corresponded to the 91 percentile of the random distribution). Although our validation panel included too few individuals to offer definitive evidence, our targeted sequencing experiment supports the idea that the detected polymorphism upstream of the *EDAR* coding region is associated with the extent of lateral plating.

Our Sanger sequence data further revealed that the target SNP at the *EDAR* locus was in perfect physical linkage with a 2 bp indel polymorphism just 4 bp downstream of this marker (details given in Supplementary Analysis 1). Given that the haplotype harboring the deletion is the one associated with reduced plating, it is tempting to speculate that this deletion disrupts a regulatory element enhancing the expression of the *EDAR* gene. However, the sliding window showing the strongest differentiation in the Complete^CL^ vs Partial^CL^ genome scan mapped much closer to the *EDAR* gene sequence (Fig. 3). Hence, our top-differentiation SNP and the associated indel in the *EDAR* region may not be the polymorphisms directly causally related to lateral plate variation.

### Frequency of alleles associated with reduced plating in natural populations

**Fig. 5.**
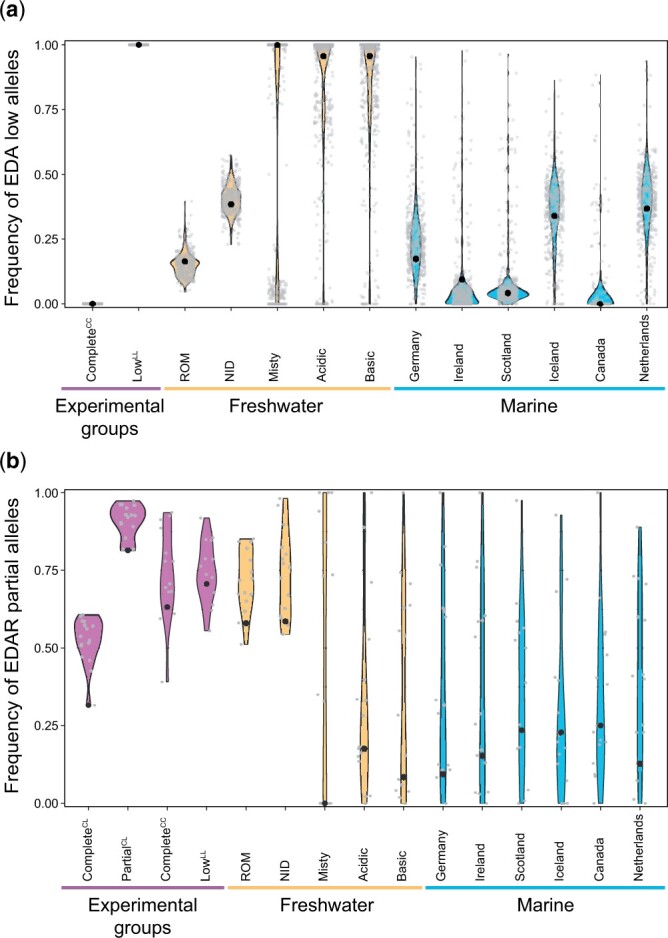
Frequency of the *EDA* low (top) and *EDAR* partial (bottom) alleles in the experimental groups, and in natural freshwater and marine stickleback populations. These alleles are associated with reduced lateral plating. In (a), the dots represent, in each sample, the 409 SNPs around the *EDA* gene showing maximal differentiation (*AFD *=* *1) in the Complete^CC^ vs Low^LL^ group comparison (see Fig. 2a bottom). One of these SNPs, located in immediate proximity to the Stn382 marker used for *EDA* genotyping, is highlighted as larger black dot. In (b), the dots represent the 16 SNPs showing the strongest differentiation near the *EDAR* gene in the Complete^CL^ vs Partial^CL^ genome scan, and the larger black dot indicates the top-*AFD* SNP in this region (Fig. 3 bottom). The colored shapes (“violins”) show the smoothed kernel density of the data. The allele frequency estimates from all samples are based on pooled whole-genome sequence data.

For the *EDA* and *EDAR* loci, we explored allele frequencies in natural populations, predicting that alleles reducing plating should be rare or absent in marine stickleback under selection for complete armor, but more frequent in freshwater populations that typically evolve reduced plating. This prediction was supported for the *EDA* locus (Fig. 5a): apart from the Lake Constance population (ROM) known to display a pelagic life style and to be selected for complete armor like marine stickleback (Lucek *et al.* 2012b; Moser *et al.* 2012; Roesti *et al.* 2015), freshwater populations tended to exhibit a higher frequency of alleles associated with the *EDA* low morph than the marine samples. Nevertheless, at least in some marine populations, the *EDA* low alleles occurred in appreciable frequencies, confirming that the genetic factor favorable in freshwater is generally available as standing genetic variation (e.g. Colosimo *et al.* 2005; Terekhanova *et al.* 2014).

At the *EDAR* locus too, marine stickleback consistently displayed a relatively low frequency of the partial allele at the top-differentiation SNP and most of the surrounding high-differentiation SNPs (Fig. 5b). However, the same also held for all inspected freshwater populations from outside the Lake Constance basin. Assuming that a polymorphism at the *EDAR* locus is truly a driver of lateral plating in stickleback, the occurrence of the *EDAR* partial allele at relatively low frequency in most of the inspected freshwater populations may be explained by dominance at the major plate locus *EDA*. Selection for reduced plating in freshwater is strong and generally results in the rapid fixation of the *EDA* low allele (Bell *et al.* 2004; Terekhanova 2014; Lescak *et al.* 2015), hence freshwater individuals are generally homozygous for the *EDA* low allele (see Fig. 5a). However, our study suggests that *EDAR* polymorphism has an effect on lateral plating in individuals heterozygous at *EDA* only. As this *EDA* genotype rapidly becomes rare during freshwater adaptation, the opportunity for selection of the *EDAR* partial allele in freshwater may often be quite limited. In contrast, in marine populations in which *EDA* heterozygotes may be more common (Fig. 5a), selection against the *EDAR* partial allele might be more effective. Nevertheless, our marine allele frequency data indicate that *EDAR* polymorphism is still widespread as standing genetic variation within the ancestral habitat.

### Conclusions

Our study indicates polymorphism at the *EDAR* locus, a member of the ectodysplasin signaling pathway, as a new candidate factor influencing lateral plating in a European stickleback population. Future work in this system, and in other populations also showing a wide range of plate phenotypes, is now needed for a definitive evaluation of the proposed phenotypic effect of *EDAR*. If a causative role of *EDAR* is confirmed, estimating this locus’ effect size using individual-level sequence data, and elucidating in which genetic backgrounds and under which ecological conditions this polymorphism is selectively relevant, are avenues for future research. Combined with the genome scan data from the *EDA* locus, our study also highlights the physical mapping resolution achieved when exploiting historical recombination via pooled whole-genome sequencing of targeted experimental groups derived from natural population samples.

## Data availability

All raw whole-genome sequence data are available from the NCBI sequence read archive (SRA) under the study number SRP222265 and the accession numbers listed by sample in a file on the Dryad repository (https://doi.org/10.6078/D1VD86). All code and supplementary data files allowing full replication of the study are available from Dryad under the same link.

Supplemental material is available at *G3* online.

## Supplementary Material

jkac077_Supplementary_Data

## References

[jkac077-B1] Arendt J , Reznick D. Convergence and parallelism reconsidered: what have we learned about the genetics of adaptation? Trends Ecol Evol. 2008;23(1):26–32. doi:10.1016/j.tree.2007.09.011. 18022278

[jkac077-B2] Barrett RDH , Schluter D. Adaptation from standing genetic variation. Trends Ecol Evol. 2008;23(1):38–44. 18006185 10.1016/j.tree.2007.09.008

[jkac077-B3] Bell MA , Aguirre WE, Buck NJ. Twelve years of contemporary armor evolution in a threespine stickleback population. Evolution. 2004;58(4):814–824. 15154557 10.1111/j.0014-3820.2004.tb00414.x

[jkac077-B4] Bell MA , Foster SA. The Evolutionary Biology of the Threespine Stickleback. Oxford: Oxford University; 1994.

[jkac077-B5] Bergstrom CA. Fast-start swimming performance and reduction in lateral plate number in threespine stickleback. Can J Zool. 2002;80(2):207–213. doi:10.1139/z01-226.

[jkac077-B6] Berner D. Allele frequency difference AFD - an intuitive alternative to FST for quantifying genetic population differentiation. Genes (Basel). 2019;10:308. 10.3390/genes10040308 PMC6523497 31003563

[jkac077-B7] Berner D , Ammann M, Spencer E, Rüegg A, Lüscher D, Moser D. Sexual isolation promotes divergence between parapatric lake and stream stickleback. J Evol Biol. 2017;30(2):401–411. doi:10.1111/jeb.13016. 27862535

[jkac077-B8] Berner D , Moser D, Roesti M, Buescher H, Salzburger W. Genetic architecture of skeletal evolution in European lake and stream stickleback. Evolution. 2014;68(6):1792–1805. 24571250 10.1111/evo.12390

[jkac077-B9] Berner D , Roesti M. Genomics of adaptive divergence with chromosome-scale heterogeneity in crossover rate. Mol Ecol. 2017;26(22):6351–6369. 28994152 10.1111/mec.14373

[jkac077-B10] Berner D , Roesti M, Bilobram S, Chan SK, Kirk H, Pandoh P, Taylor GA, Zhao Y, Jones SJM, DeFaveri J, et al De novo sequencing, assembly, and annotation of four threespine stickleback genomes based on microfluidic partitioned DNA libraries. Genes (Basel). 2019;10(6):426. 10.3390/genes10060426 PMC6627416 31163709

[jkac077-B11] Berner D , Roesti M, Hendry AP, Salzburger W. Constraints on speciation suggested by comparing lake-stream stickleback divergence across two continents. Mol Ecol. 2010;19(22):4963–4978. 20964754 10.1111/j.1365-294X.2010.04858.x

[jkac077-B12] Bissegger M , Laurentino TG, Roesti M, Berner D. Widespread intersex differentiation across the stickleback genome – the signature of sexually antagonistic selection? Mol Ecol. 2020;29(2):262–271. 31574563 10.1111/mec.15255

[jkac077-B13] Chan YF , Marks ME, Jones FC, Villarreal G, Shapiro MD, Brady SD, Southwick AM, Absher DM, Grimwood J, Schmutz J, et al Adaptive evolution of pelvic reduction in sticklebacks by recurrent deletion of a Pitx1 enhancer. Science. 2010;327(5963):302–305. doi:10.1126/science.1182213. 20007865 PMC3109066

[jkac077-B14] Cheng J , Sedlazek F, Altmüller J, Nolte AW. Ectodysplasin signalling genes and phenotypic evolution in sculpins (*Cottus*). Proc R Soc B. 2015;282(1815):20150746.doi:10.1098/rspb.2015.0746. PMC4614746 26354934

[jkac077-B15] Cleves PA , Hart JC, Agoglia RM, Jimenez MT, Erickson PA, Gai L, Miller CT. An intronic enhancer of Bmp6 underlies evolved tooth gain in sticklebacks. PLoS Genet. 2018;14(6):e1007449.doi:10.1371/journal.pgen.1007449. 29902209 PMC6019817

[jkac077-B16] Colosimo PF , Peichel CL, Nereng K, Blackman BK, Shapiro MD, Schluter D, Kingsley DM. The genetic architecture of parallel armor plate reduction in threespine sticklebacks. PLoS Biol. 2004;2(5):e109. 15069472 10.1371/journal.pbio.0020109 PMC385219

[jkac077-B17] Colosimo PF , Hosemann KE, Balabhadra S, Villarreal G, Dickson M, Grimwood J, Schmutz J, Myers RM, Schluter D, Kingsley DM, et al Widespread parallel evolution in sticklebacks by repeated fixation of ectodysplasin alleles. Science. 2005;307(5717):1928–1933. 15790847 10.1126/science.1107239

[jkac077-B18] Cresko WA , Amores A, Wilson C, Murphy J, Currey M, Phillips P, Bell MA, Kimmel CB, Postlethwait JH. Parallel genetic basis for repeated evolution of armor loss in Alaskan threespine stickleback populations. Proc Natl Acad Sci U S A. 2004;101(16):6050–6055. 15069186 10.1073/pnas.0308479101 PMC395921

[jkac077-B19] Cui CY , Schlessinger D. EDA signaling and skin appendage development. Cell Cycle. 2006;5(21):2477–2483. doi:10.4161/cc.5.21.3403. 17102627 PMC2860309

[jkac077-B20] Ferretti L , Ramos-Onsins SE, Pérez-Enciso M. Population genomics from pool sequencing. Mol Ecol. 2013;22(22):5561–5576. doi:10.1111/mec.12522. 24102736

[jkac077-B21] Flaxman SM , Wacholder AC, Feder JL, Nosil P. Theoretical models of the influence of genomic architecture on the dynamics of speciation. Mol Ecol. 2014;23(16):4074–4088. doi:10.1111/mec.12750. 24724861

[jkac077-B22] Galloway J , Cresko WA, Ralph P. A few stickleback suffice for the transport of alleles to new lakes. G3 (Bethesda). 2020;10(2):505–514. doi:10.1101/713040. 31801796 PMC7003093

[jkac077-B23] Gautier M , Foucaud J, Gharbi K, Cézard T, Galan M, Loiseau A, Thomson M, Pudlo P, Kerdelhué C, Estoup A, et al Estimation of population allele frequencies from next-generation sequencing data: pool-versus individual-based genotyping. Mol Ecol. 2013;22(14):3766–3779. doi:10.1111/mec.12360. 23730833

[jkac077-B24] Glazer AM , Killingbeck EE, Mitros T, Rokhsar DS, Miller CT. Genome assembly improvement and mapping convergently evolved skeletal traits in sticklebacks with genotyping-by-sequencing. G3 (Bethesda). 2015;5(7):1463–1472. doi:10.1534/g3.115.017905. 26044731 PMC4502380

[jkac077-B25] Haenel Q , Guerard L, MacColl ADC, Berner D. The maintenance of standing genetic variation: Gene flow vs. selective neutrality in Atlantic stickleback fish. Mol Ecol. 2022;31(3):811–821. 10.1111/mec.16269 34753205 34753205 PMC9299253

[jkac077-B26] Haenel Q , Oke KB, Laurentino TG, Hendry AP, Berner D. Clinal genomic analysis reveals strong reproductive isolation across a steep habitat transition in stickleback fish. Nat Commun. 2021;12(1):4850. doi:10.1101/2020.08.28.269753. 34381033 PMC8358029

[jkac077-B27] Harris MP , Rohner N, Schwarz H, Perathoner S, Konstantinidis P, Nüsslein-Volhard C. Zebrafish EDA and EDAR mutants reveal conserved and ancestral roles of ectodysplasin signaling in vertebrates. PLoS Genet. 2008;4(10):e1000206.doi:10.1371%2Fjournal.pgen.1000206. 18833299 10.1371/journal.pgen.1000206 PMC2542418

[jkac077-B28] Hereford J. A quantitative survey of local adaptation and fitness trade-offs. Am Nat. 2009;173(5):579–588. doi:10.1086/597611. 19272016

[jkac077-B29] Howes TR , Summers BR, Kingsley DM. Dorsal spine evolution in threespine sticklebacks via a splicing change in MSX2A. BMC Biol. 2017;15(1):115.doi:10.1186/s12915-017-0456-5. 29212540 PMC5719529

[jkac077-B30] Iida Y , Hibiya K, Inohaya K, Kudo A. EDA/EDAR signaling guides fin ray formation with preceding osteoblast differentiation, as revealed by analyses of the medaka all-fin less mutant afl. Dev Dyn. 2014;243(6):765–777. doi:10.1002/dvdy.24120. 24585696

[jkac077-B31] Indjeian VB , Kingman GA, Jones FC, Guenther CA, Grimwood J, Schmutz J, Myers RM, Kingsley DM. Evolving new skeletal traits by cis-regulatory changes in bone morphogenetic proteins. Cell. 2016;164(1–2):45–56. doi:10.1016/j.cell.2015.12.007. 26774823 PMC4759241

[jkac077-B32] Jones FC , Grabherr MG, Chan YF, Russell P, Mauceli E, Johnson J, Swofford R, Pirun M, Zody MC, White S, et al; Broad Institute Genome Sequencing Platform & Whole Genome Assembly Team. The genomic basis of adaptive evolution in threespine sticklebacks. Nature. 2012;484(7392):55–61. doi:10.1038/nature10944. 22481358 PMC3322419

[jkac077-B33] Knecht AK , Hosemann KE, Kingsley DM. Constraints on utilization of the EDA-signaling pathway in threespine stickleback evolution. Evol Dev. 2007;9(2):141–154. doi:10.1111/j.1525-142X.2007.00145.x. 17371397 10.1111/j.1525-142X.2007.00145.x

[jkac077-B34] Kondo S , Kuwahara Y, Kondo M, Naruse K, Mitani H, Wakamatsu Y, Ozato K, Asakawa S, Shimizu N, Shima A, et al The medaka rs-3 locus required for scale development encodes ectodysplasin-A receptor. Curr Biol. 2001;11(15):1202–1206. doi:10.1016/S0960-9822(01)00324-4. 11516953 10.1016/s0960-9822(01)00324-4

[jkac077-B35] Kristjánsson B. Rapid morphological changes in threespine stickleback, *Gasterosteus aculeatus*, in freshwater. Environ Biol Fish. 2005;74(3–4):357–363. doi:10.1007/s10641-005-1487-2.

[jkac077-B36] Laurentino TG , Moser D, Roesti M, Ammann M, Frey A, Ronco F, Kueng B, Berner D. Genomic release-recapture experiment in the wild reveals within-generation polygenic selection in stickleback fish. Nat Commun. 2020;11(1):1928.doi:10.1038/s41467-020-15657-3. 32317640 PMC7174299

[jkac077-B37] Leimu R , Fischer MC. A meta-analysis of local adaptation in plants. PLoS One. 2008;3(12):e4010.doi:10.1371/journal.pone.0004010. 19104660 PMC2602971

[jkac077-B38] Leinonen T , Herczeg G, Cano JM, Merilä J. Predation-imposed selection on threespine stickleback (*Gasterosteus aculeatus*) morphology: a test of the refuge use hypothesis. Evolution. 2011;65(10):2916–2926. doi:10.1111/j.1558-5646.2011.01349.x. 21967432

[jkac077-B39] Le Rouzic A , Østbye K, Klepaker TO, Hansen TF, Bernatchez L, Schluter D, Vøllestad LA. Strong and consistent natural selection associated with armour reduction in sticklebacks. Mol Ecol. 2011;20(12):2483–2493. doi:10.1111/j.1365-294X.2011.05071.x. 21443674

[jkac077-B40] Lescak EA , Bassham SL, Catchen J, Gelmond O, Sherbick ML, von Hippel FA, Cresko WA. Evolution of stickleback in 50 years on earthquake-uplifted islands. Proc Natl Acad Sci U S A. 2015;112(52):E7204–E7212. doi:10.1073/pnas.1512020112. 26668399 PMC4702987

[jkac077-B41] Lucek K , Haesler MP, Sivasundar A. When phenotypes do not match genotypes—unexpected phenotypic diversity and potential environmental constraints in Icelandic stickleback. J Hered. 2012a;103(4):579–584. doi:10.1093/jhered/ess021. 22563124

[jkac077-B42] Lucek K , Sivasundar A, Seehausen O. Evidence of adaptive evolutionary divergence during biological invasion. PLoS One. 2012b;7(11):e49377. 23152900 10.1371/journal.pone.0049377 PMC3495884

[jkac077-B43] Marques DA , Lucek K, Meier JI, Mwaiko S, Wagner CE, Excoffier L, Seehausen O. Genomics of rapid incipient speciation in sympatric threespine stickleback. PLoS Genet. 2016;12(2):e1005887. 26925837 10.1371/journal.pgen.1005887 PMC4771382

[jkac077-B44] Martin A , Orgogozo V. The loci of repeated evolution: a catalog of genetic hotspots of phenotypic variation. Evolution. 2013;67(5):1235–1250. doi:10.1111/evo.12081. 23617905

[jkac077-B45] Messer PW , Petrov DA. Population genomics of rapid adaptation by soft selective sweeps. Trends Ecol Evol. 2013;28(11):659–669. doi:10.1016/j.tree.2013.08.003. 24075201 PMC3834262

[jkac077-B46] Mikkola ML , Thesleff I. Ectodysplasin signaling in development. Cytokine Growth Factor Rev. 2003;14(3–4):211–224. doi:10.1016/s1359-6101(03)00020-0. 12787560 10.1016/s1359-6101(03)00020-0

[jkac077-B47] Miller CT , Beleza S, Pollen AA, Schluter D, Kittles RA, Shriver MD, Kingsley DM. Cis-regulatory changes in kit ligand expression and parallel evolution of pigmentation in sticklebacks and humans. Cell. 2007;131(6):1179–1189. 18083106 10.1016/j.cell.2007.10.055 PMC2900316

[jkac077-B48] Morgan M , Pages H, Obenchain V, Hayden N. Rsamtools: Binary Alignment (BAM), FASTA, Variant Call (BCF), and tabix file import. R Package, version 1.3.0. 2017. http://Bioco nduct or.Org/Packa ges/Release/Bioc/Html/Rsamt ools.Html

[jkac077-B49] Moser D , Roesti M, Berner D. Repeated lake-stream divergence in stickleback life history within a Central European Lake Basin. PLoS One. 2012;7(12):e50620. doi:10.1371%2Fjournal.pone.0050620. 23226528 10.1371/journal.pone.0050620 PMC3514289

[jkac077-B50] Mousseau TA , Sinervo B, Endler JA. Adaptive Genetic Variation in the Wild. New York (NY): Oxford University; 2000.

[jkac077-B51] Nelson TC , Cresko WA. Ancient genomic variation underlies repeated ecological adaptation in young stickleback populations. Evol Lett. 2018;2(1):9–21. doi:10.1002/evl3.37. 30283661 PMC6121857

[jkac077-B52] O'Brown NM , Summers BR, Jones FC, Brady SD, Kingsley DM. A recurrent regulatory change underlying altered expression and Wnt response of the stickleback armor plates gene EDA. Elife. 2015;4:e05290.doi:10.7554/eLife.05290. 25629660 PMC4384742

[jkac077-B53] Ralph P , Coop G. Parallel adaptation: one or many waves of advance of an advantageous allele? Genetics. 2010;186(2):647–668. doi:10.1534/genetics.110.119594. 20660645 PMC2954473

[jkac077-B54] Reimchen TE. Injuries on stickleback from attacks by a toothed predator (*Oncorhynchus*) and implication for the evolution of lateral plates. Evolution (NY). 1992;46(4):1224–1230. 10.1111/j.1558-5646.1992.tb00631.x 28564400

[jkac077-B55] Reimchen TE. Predator handling failures of lateral plate morphs in *Gasterosteus aculeatus*: functional implications for the ancestral plate condition. Behaviour. 2000;137(7–8):1081–1096.

[jkac077-B56] Roesti M , Gavrilets S, Hendry AP, Salzburger W, Berner D. The genomic signature of parallel adaptation from shared genetic variation. Mol Ecol. 2014;23(16):3944–3956. 24635356 10.1111/mec.12720 PMC4122612

[jkac077-B57] Roesti M , Kueng B, Moser D, Berner D. The genomics of ecological vicariance in threespine stickleback fish. Nat Commun. 2015;6:8767.doi:10.1038/ncomms9767. 26556609 PMC4659939

[jkac077-B58] Roesti M , Salzburger W, Berner D. Uninformative polymorphisms bias genome scans for signatures of selection. BMC Evol Biol. 2012;12:94. 22726891 10.1186/1471-2148-12-94 PMC3426483

[jkac077-B59] Schluter D. The Ecology of Adaptive Radiation. Oxford: Oxford University; 2000.

[jkac077-B60] Shono T , Thiery AP, Cooper RL, Kurokawa D, Britz R, Okabe M, Fraser GJ. Evolution and developmental diversity of skin spines in Pufferfishes. iScience. 2019;19:1248–1255. doi:10.1016/j.isci.2019.06.003. 31353167 PMC6831732

[jkac077-B61] Stoler N , Nekrutenko A. Sequencing error profiles of Illumina sequencing instruments. NAR Genom Bioinform. 2021;3(1):lqab019.doi:10.1093/nargab/lqab019 33817639 PMC8002175

[jkac077-B62] Terekhanova NV , Logacheva MD, Penin AA, Neretina TV, Barmintseva AE, Bazykin GA, Kondrashov AS, Mugue NS. Fast evolution from precast bricks: genomics of young freshwater populations of threespine stickleback *Gasterosteus aculeatus*. PLoS Genet. 2014;10(10):e1004696.doi:10.1371/journal.pgen.1004696. 25299485 10.1371/journal.pgen.1004696 PMC4191950

[jkac077-B63] Thompson KA , Osmond MM, Schluter D. Parallel genetic evolution and speciation from standing variation. Evol Lett. 2019;3(2):129–141. doi:10.1002/evl3.106. 31289688 PMC6591551

[jkac077-B64] Villoutreix R , Ayala D, Joron M, Gompert Z, Feder JL, Nosil P. Inversion breakpoints and the evolution of supergenes. Mol Ecol. 2021;30(12):2738–2755. doi:10.1111/mec.15907. 33786937 PMC7614923

[jkac077-B65] Wucherpfennig JI , Miller CT, Kingsley DM. Efficient CRISPR-Cas9 editing of major evolutionary loci in sticklebacks. Evol Ecol Res. 2019;20(1):107–132. 34899072 PMC8664273

[jkac077-B66] Yamasaki YY , Mori S, Kokita T, Kitano J. Armour plate diversity in Japanese freshwater threespine stickleback (*Gasterosteus aculeatus*). Evol Ecol Res. 2019;20:51–67.

[jkac077-B67] Yeaman S. Local adaptation by alleles of small effect. Am Nat. 2015;186(S1):S74–S89. doi:10.1086/682405. 26656219

