# Supplementary material for: The ectodysplasin-A receptor is a candidate gene for lateral plate number variation in stickleback fish
Source: G3 (Bethesda). 2022 Apr 4;12(6):jkac077. doi: 10.1093/g3journal/jkac077 (PMC9157104; doi:10.1093/g3journal/jkac077)
Supplement: jkac077_Supplementary_Data [file jkac077_supplementary_data.pdf]

Supplementary Information to

The EDA receptor (EDAR) is a candidate gene for lateral plate number variation in stickleback fish

by Telma G. Laurentino, Nico Boileau, Fabrizia Ronco, Daniel Berner

Contents

- **Analysis S1** Sanger sequencing of the EDAR locus in the validation panel **p. 2**
- **Figure S1** Justification of read depth cutoffs used for SNP calling **p. 4**
- **Figure S2** Differentiation between Complete^{CC} and Low^{LL} across all chromosomes **p. 5**
- **Figure S3** Differentiation between Complete^{CL} and Partial^{CL} across all chromosomes **p. 8**
- **Figure S4** Concordance in differentiation at the EDAR locus between two independent genome assemblies **p. 11**
- **Figure S5** Gene annotation and functional evaluation of the regions containing the ten top-AFD SNPs in the Complete^{CL} vs. Partial^{CL} comparison **p. 12**
- **Supplementary Literature** **p. 14**

Analysis S1

Exploration of an association between EDAR-associated alleles and the partial lateral plate morph using individual Sanger sequencing

Our sequencing considered a 'validation panel' of 46 individuals from the Lake Constance basin not used for the main genome scans. The individuals and their genotypes are described in the table below. PCR was conducted using the forward primer `GTTTCTCGGACATGGCTTTTATG` and the reverse primer `GTCACTACACACAGCACACA` (both at 10 uM), producing a 247 (or 245) bp amplicon. The PCR used RedTaq and the following amplification conditions: Initialization 94°C for 3 minutes; cycling (35x) 94°C for 20 seconds, 56°C for 20 seconds and 72°C for 20 seconds; final elongation 42°C for 4 minutes. The product was sequenced on an ABI3130xl sequencer (Applied Biosystems).

The sequence below shows the primer binding sites (forward shaded blue, reverse shaded yellow), the position of the top-AFD SNP near the EDAR gene (shaded gray) and an associated 2-bp indel (purple). The two haplotypes associated with partial and complete plating are also visualized. Across our validation panel, the SNP and indel alleles showed perfect linkage.

```
5'_GTTTCTCGGACATGGCTTTTATGTATTGAAATCAATATTCTTAGAAGGTAACTGTGAAATCTGGATTGT
TTGATTGTTGACACAAACTGTGGTGCTCGGGTGTACTTTCTGAGCGTGCTGTGGTCTTATCTGGGCGTCCC
TCTAAAGTTAGCTGTTGGGAGCGGATCGCTACCCTACAAACGCCTCCCGTCCCTCCTGAAAGCAGTCAGAA
ACTTTCCCATGCTCTCTGTGTGCTGTGTGTAGTGAC_3'
```

Partial plating haplotype: `GTCCTTCT--AGTTA`
Complete plating haplotype: `GTCCCTCTAAAGTTA`

Specimen ID	Population	Genotype (P=partial allele, C=complete allele)
Ga1112	Lake Constance	PP
Ga1114	Lake Constance	CP
Ga1130	Lake Constance	PP
Ga1920	NID stream	PP
Ga1941	NID stream	CP
Ga1961	NID stream	CC
Ga1977	NID stream	CP
Ga2008	NID stream	PP
Ga2028	NID stream	CP
Ga2031	NID stream	PP
Ga2034	NID stream	CP
Ga2036	NID stream	PP
Ga2037	NID stream	CP
Ga2049	NID stream	CP
Ga2065	NID stream	CP
Ga3168	F2hybrids	CP
Ga3182	F2hybrids	PP
Ga3184	F2hybrids	CP
Ga3189	F2hybrids	PP

Ga3232	F2hybrids	CP
Ga3259	F2hybrids	CP
Ga3282	F2hybrids	CP
Ga3284	F2hybrids	CP
Ga3364	F2hybrids	PP
Ga3370	F2hybrids	CP
Ga3371	F2hybrids	CP
Ga3428	F2hybrids	CP
Ga3431	F2hybrids	PP
Ga3432	F2hybrids	CP
Ga3435	F2hybrids	CP
Ga3466	F2hybrids	CP
Ga3475	F2hybrids	PP
Ga3506	F2hybrids	PP
Ga3549	F2hybrids	CP
Ga4054	Lake Constance	PP
Ga4055	Lake Constance	CP
Ga4056	Lake Constance	PP
Ga4057	Lake Constance	CP
Ga4058	Lake Constance	PP
Ga4059	Lake Constance	CP
Ga4060	Lake Constance	CC
Ga4061	Lake Constance	PP
Ga4062	Lake Constance	CP
Ga4063	Lake Constance	CP
Ga4064	Lake Constance	CP
Ga4065	Lake Constance	CP

Figure S1

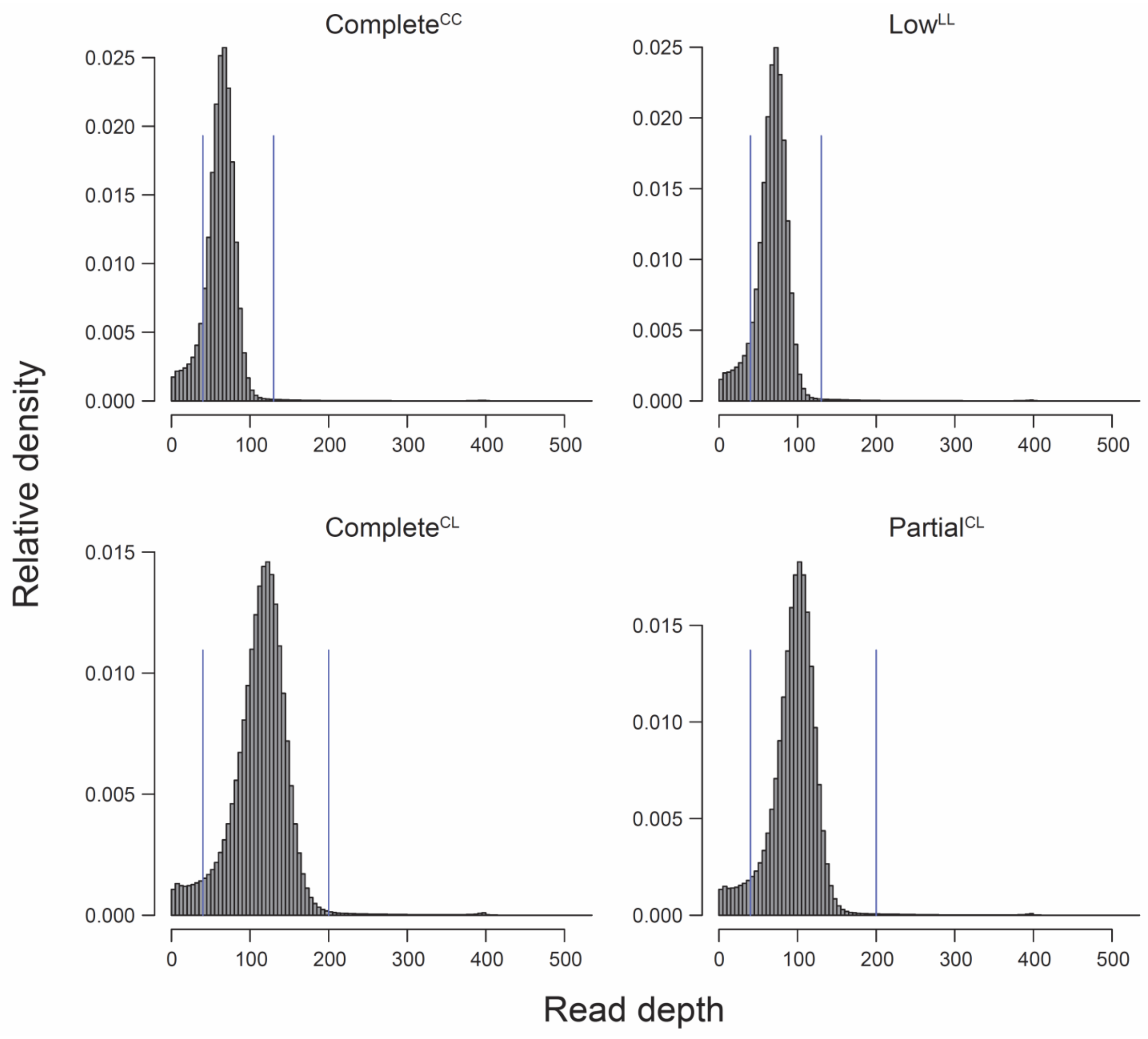

Figure S1 Read depth thresholds used for SNP calling. In the Complete^{CC} vs. Low^{LL} comparison, a read depth between 40 and 130x was required within each group for a SNP to be accepted for further analysis. In the Complete^{CL} vs. Partial^{CL} comparison, the thresholds were 40 and 200x. The histograms show the genome-wide distribution of read depth per base position for each of the four groups. The blue vertical lines indicate the read depth thresholds applied to each group. The upper read depth thresholds were chosen visually to exclude repeated genomic regions with excessive coverage. The lower read depth threshold was chosen to exclude positions at which allele frequency estimation would have been relatively imprecise (Ferretti et al. 2013; Berner 2019), although this slightly truncated the read depth distribution toward the lower end (especially in the Complete^{CC} vs. Low^{LL} comparison).

Figure S2

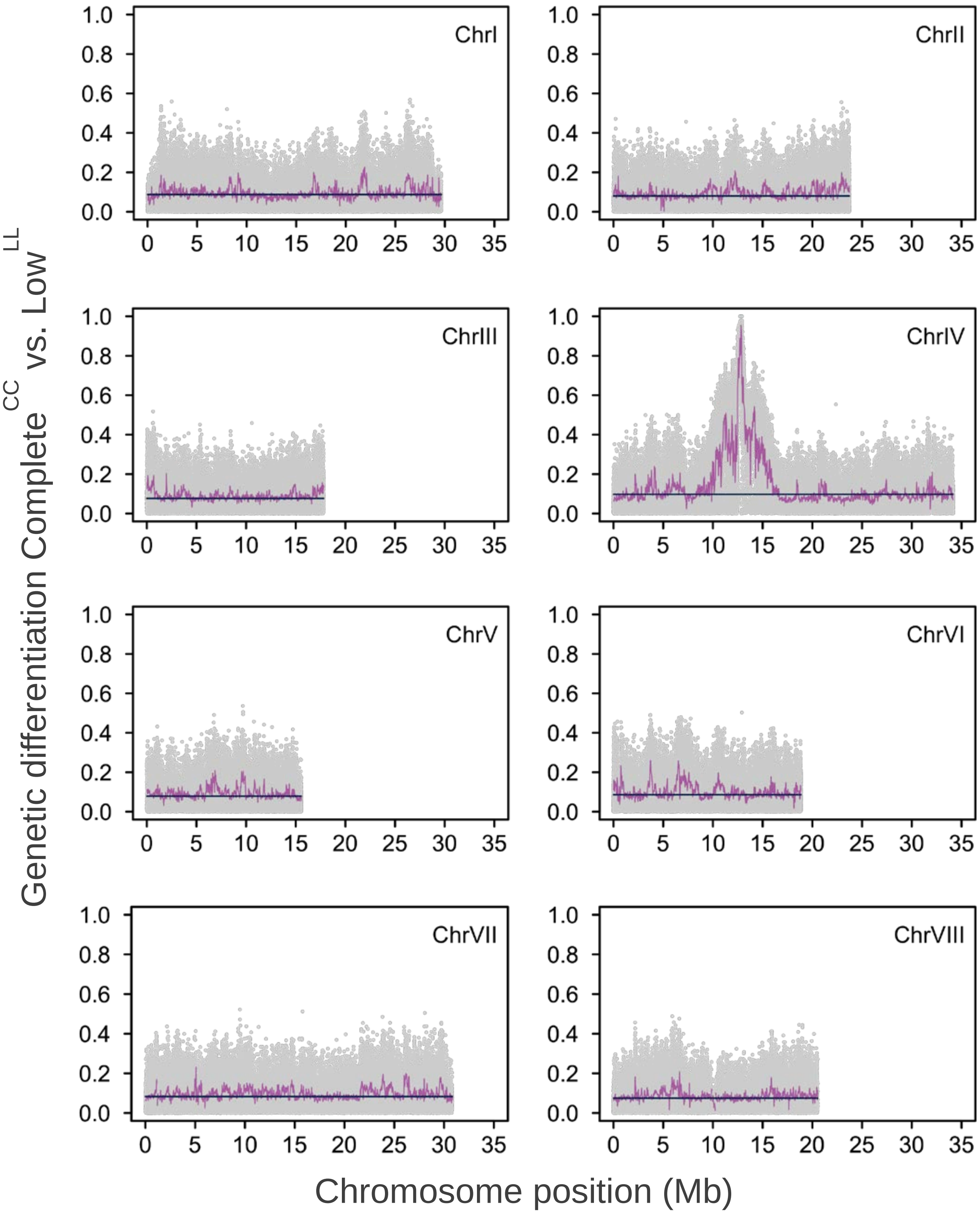
ChrI
ChrII
ChrIII
ChrIV
ChrV
ChrVI
ChrVII
ChrVIII
Genetic differentiation Complete CC vs. Low LL
Chromosome position (Mb)

Figure S2

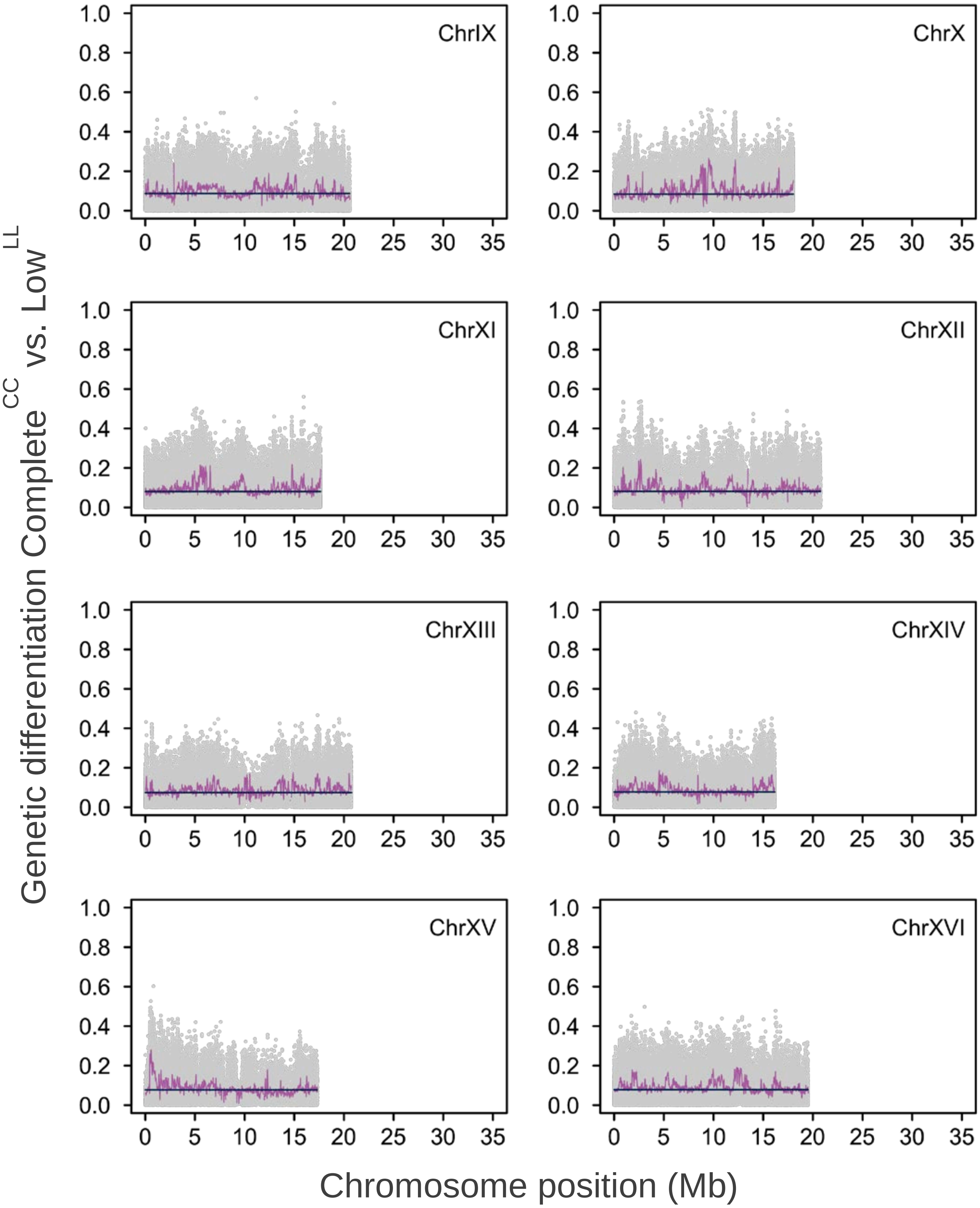
ChrIX
ChrX
ChrXI
ChrXII
ChrXIII
ChrXIV
ChrXV
ChrXVI
1.0
0.8
0.6
0.4
0.2
0.0
0 5 10 15 20 25 30 35
Genetic differentiation Complete^{CC} vs. Low^{LL}
Chromosome position (Mb)

Figure S2

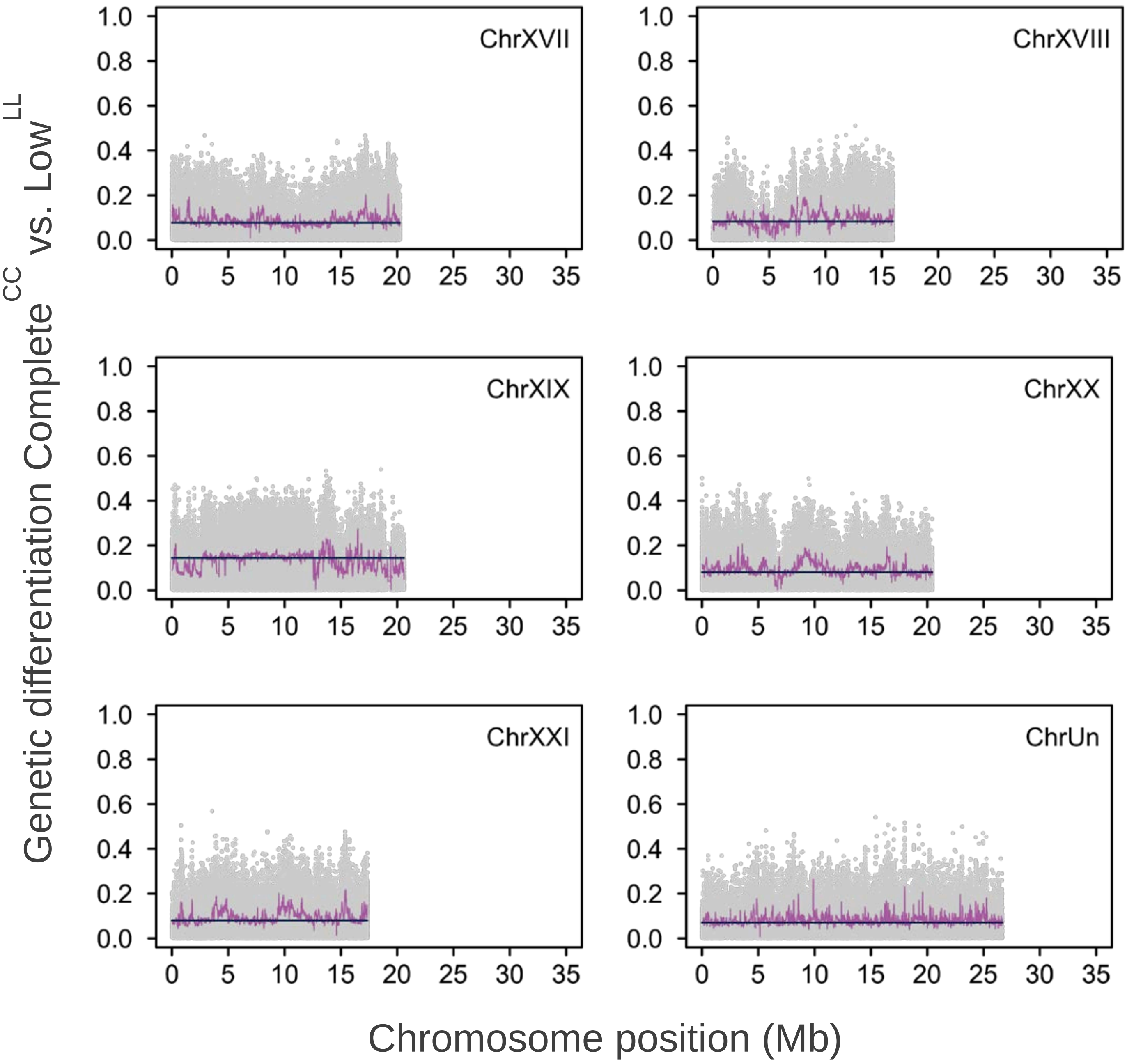

Figure S2 Genetic differentiation, quantified by the absolute allele frequency difference *AFD*, between the Complete^{CC} and Low^{LL} groups across all chromosomes. The graphing conventions follow the top panels in Figures 2 and 3.

Figure S3

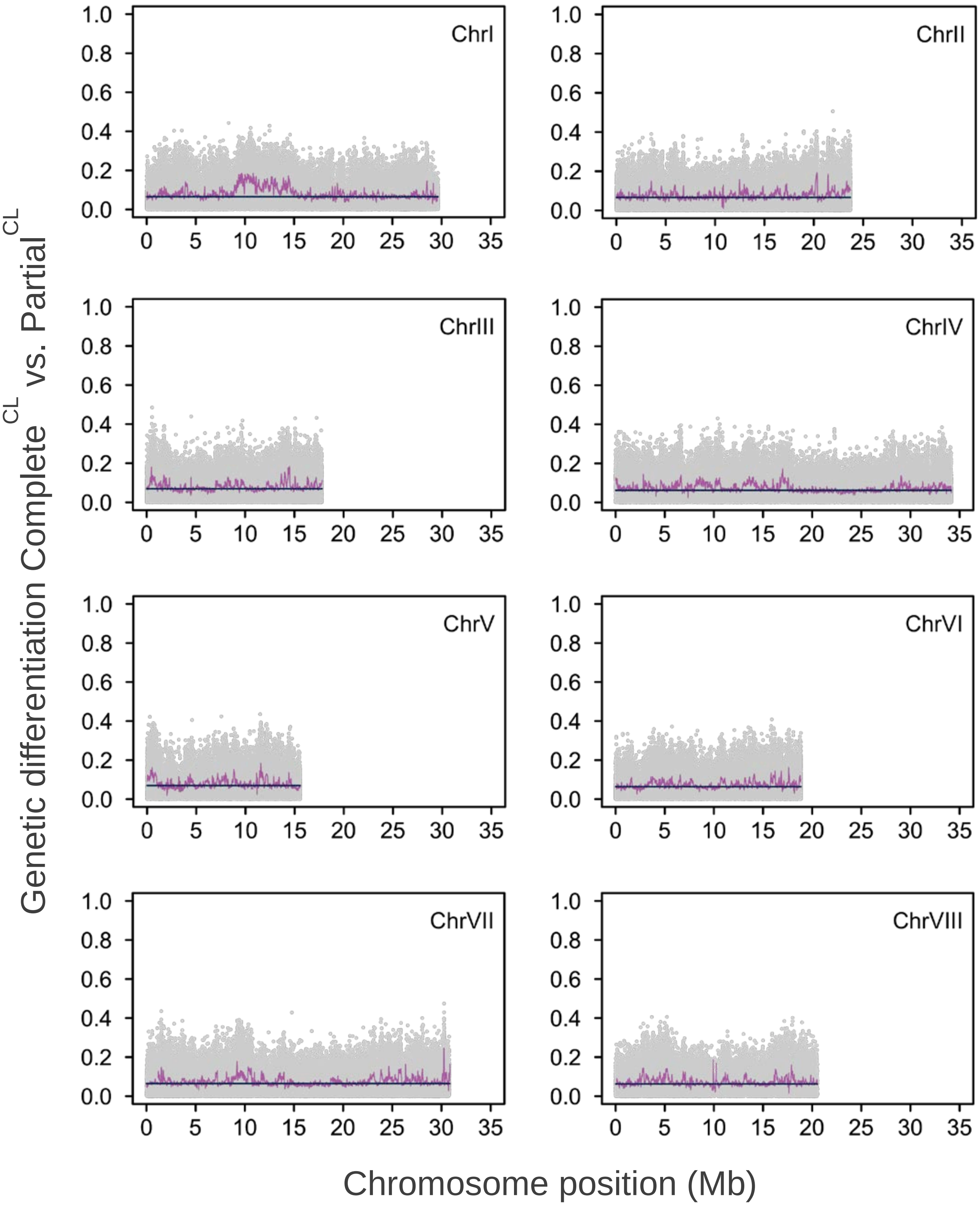
ChrI
ChrII
ChrIII
ChrIV
ChrV
ChrVI
ChrVII
ChrVIII
Genetic differentiation Complete^CL vs. Partial^CL
Chromosome position (Mb)

Figure S3

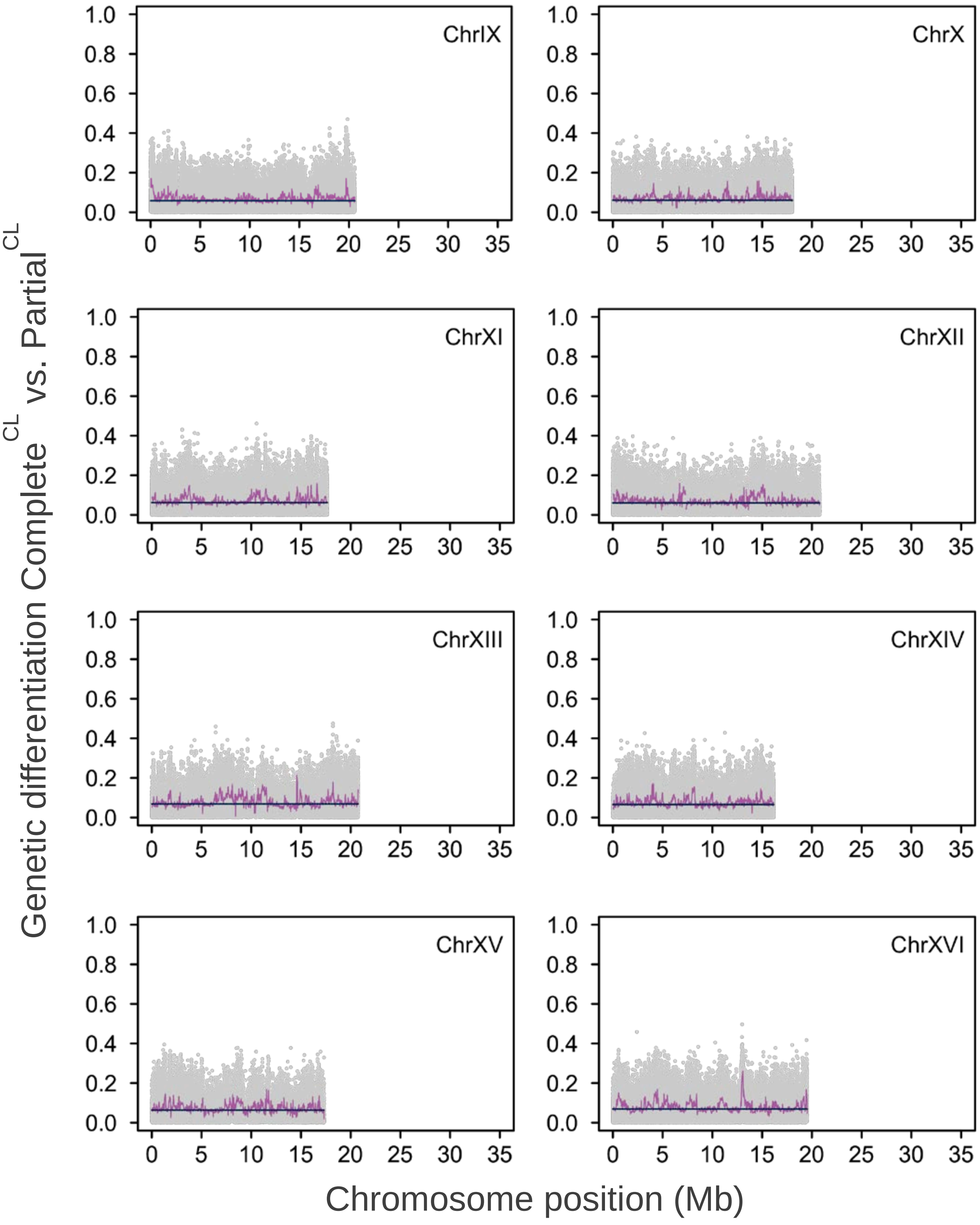
ChrIX
ChrX
ChrXI
ChrXII
ChrXIII
ChrXIV
ChrXV
ChrXVI
Genetic differentiation Complete^{CL} vs. Partial^{CL}
Chromosome position (Mb)

Figure S3

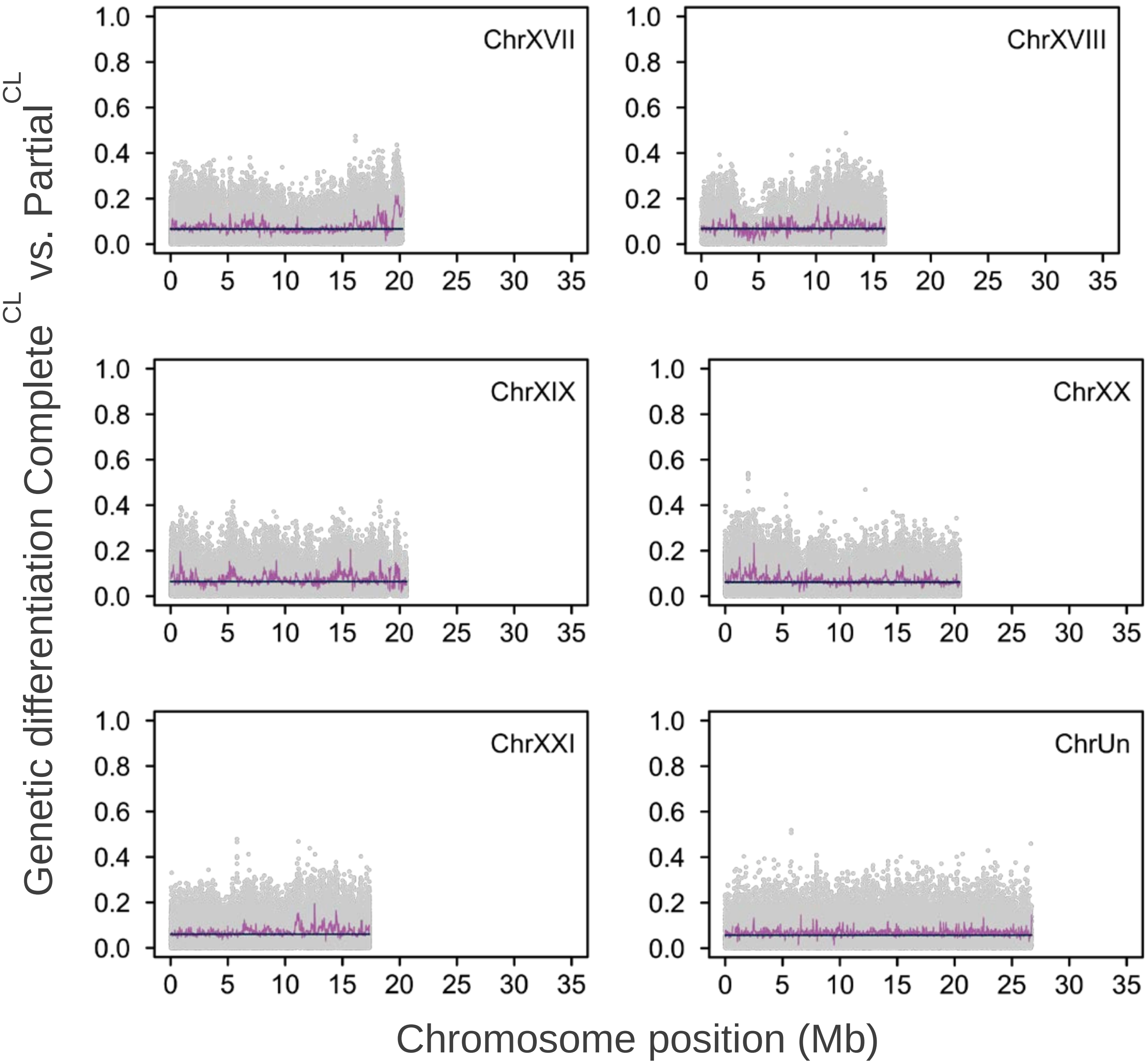

Figure S3 Genetic differentiation between the Complete[CL] and Partial[CL] groups across all chromosomes. The graphing conventions follow the top panels in Figures 2 and 3.

Figure S4

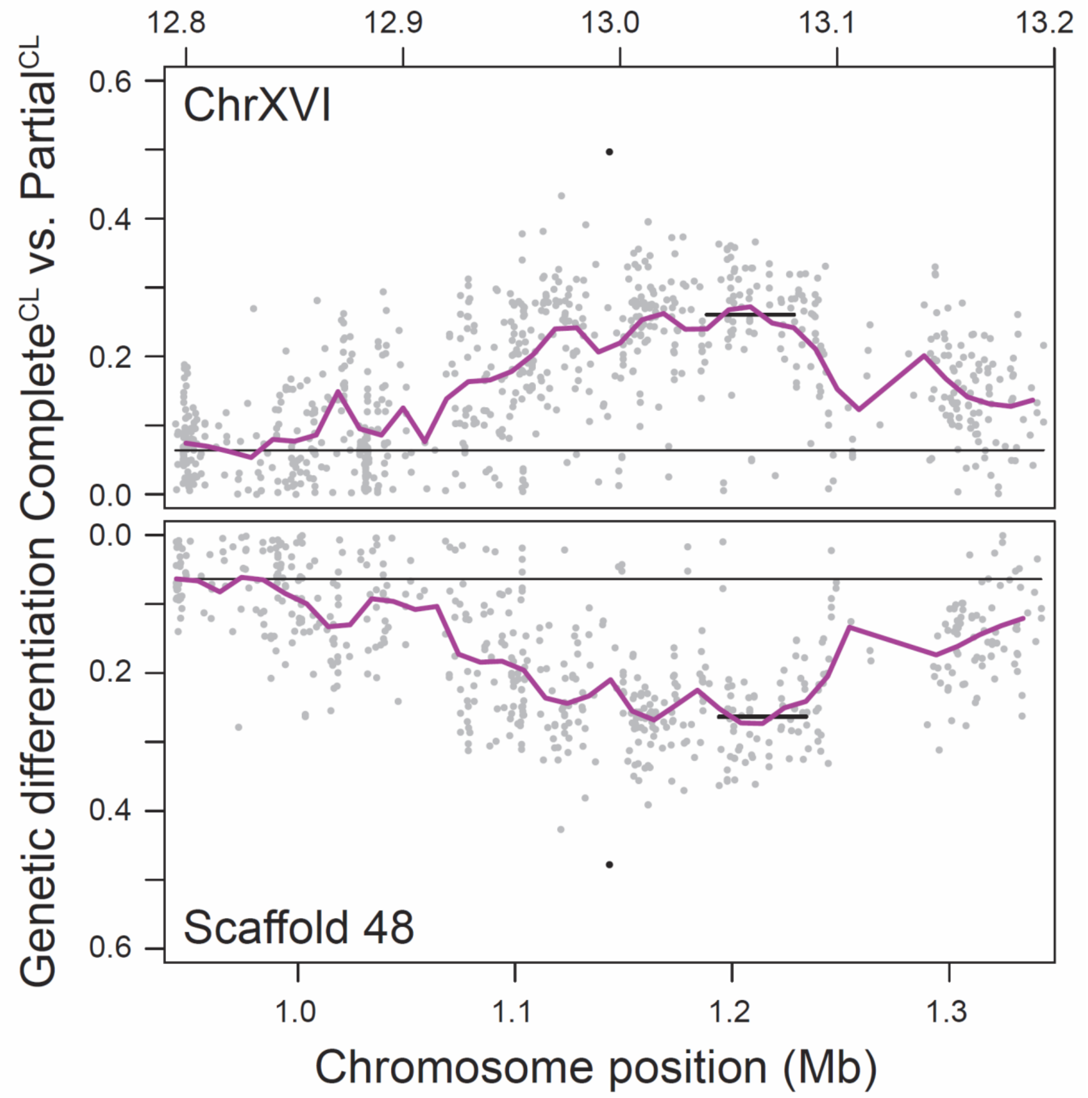

Figure S4 The DNA segment homologous to the EDAR locus within a *de novo* stickleback genome assembly based on an individual from the NID population exhibits extremely high differentiation between the Complete^{CL} and Partial^{CL} groups. The top panel is the 400 kb region also shown in Figure 3, centered on the top-AFD SNP near the EDAR gene, and based on read mapping to the third-generation stickleback genome assembly (Glazer et al. 2015). The bottom panel is the homologous segment on scaffold 48 from the Berner et al. (2019) assembly. Here the black dot represents the SNP with the second highest AFD value genome-wide, and as in the upper panel, the black horizontal bar indicates the magnitude of genetic differentiation for the 40 kb sliding window showing the strongest differentiation genome-wide. Note that the bottom panel is based on higher analytical stringency (a minimum of 60x read coverage per base position for SNP calling, as opposed to 40x).

Figure S5

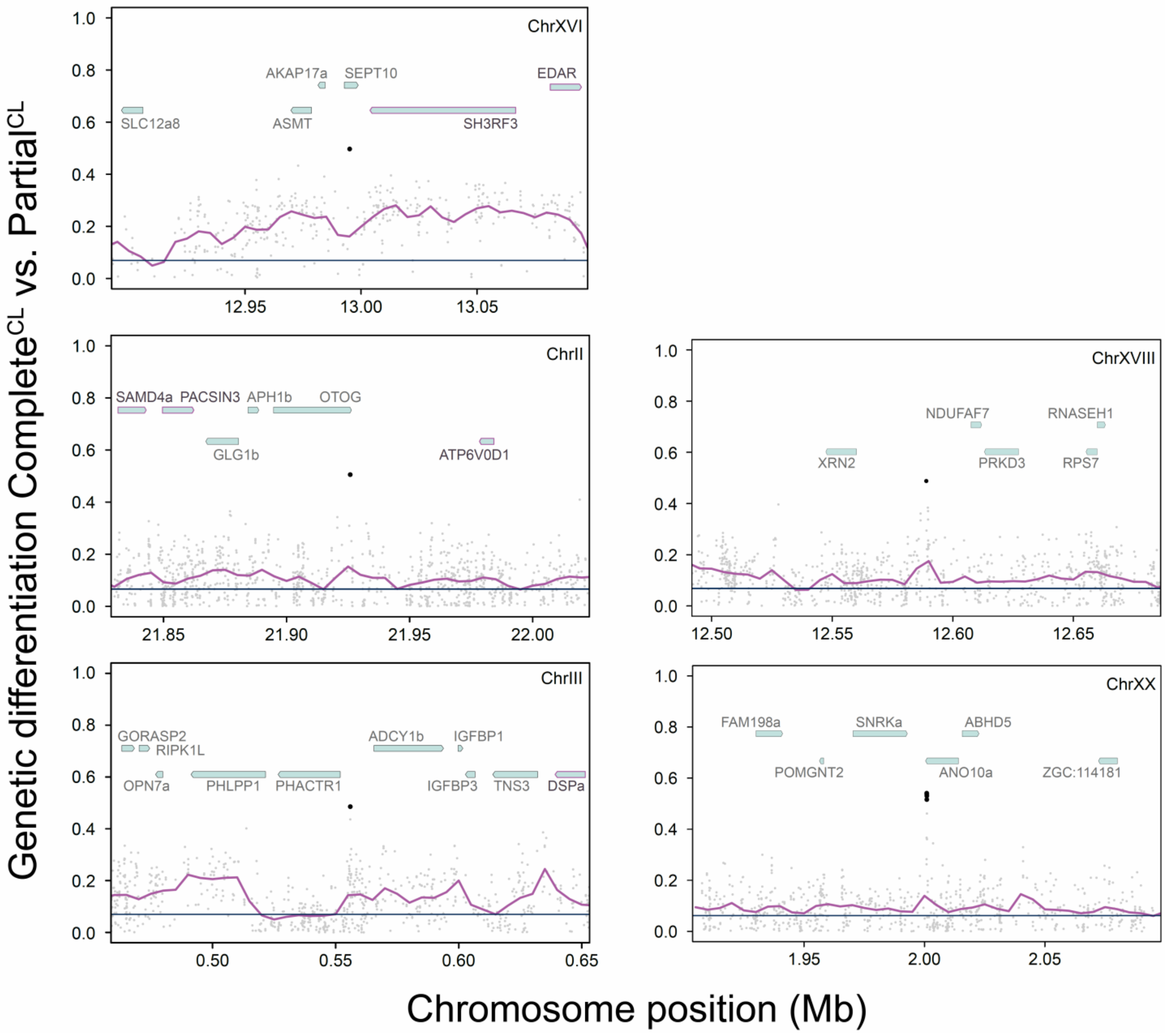

Figure S5 Gene annotation for the 180 kb regions around the SNPs showing the strongest differentiation between the Complete^{CL} and Partial^{CL} groups (black dots). Genes are shown as light blue arrows, with their direction indicating the chromosome strand, and purple borders highlighting potential functional relevance (i.e., known to be involved in bone or ectodermal development, or in the TNF or Wnt/beta-catenin pathways). Otherwise, the graphing conventions follow Figures 2 and 3. Two of the ten total high-differentiation SNPs located on scaffolds not anchored to chromosomes (i.e., located on ChrUn) are not presented. The cluster of four SNPs on ChrXX (bottom right) emerged in the main genome scan (using a relatively liberal minimum read depth threshold) only, and inspection of the nucleotide pileup confirmed read depth just passing the threshold. Hence, although the annotation for this chromosome region is presented,

the strong differentiation at these SNPs likely represents sampling stochasticity. Note that only the EDAR locus (top-left) shows substantial differentiation across a broader region around the high-AFD SNP (see also Figures 3 and S3). For this locus, methodological artifacts and sampling stochasticity can be ruled as causes of the strong differentiation between Complete^{CL} and Partial^{CL} stickleback. Combined with functional evidence on the role of EDAR in the development of the dermal skeleton in other fish species (see main text), this locus clearly emerges as the top candidate in our investigation, and was therefore subject to further analysis.

As a resource for future work, however, we here also discuss the evidence for the other annotated genes of potential functional relevance according to our criteria. On ChrXVI there is, beside EDAR, the gene SH3RF3 associated with variation in human bone mineral density (Kim 2018) and hair thickness (Adhikari et al. 2016). In the candidate region on ChrII, ATP6V-OD1 is noteworthy for its association with bone mineral density in humans (Kim 2018), while PACSIN3 has effects on the development of the fish lateral line organ (Thisse and Thisse 2004; Chen et al. 2012). The latter trait has previously been shown to be developmentally linked to lateral plates (Mills et al. 2014). A third candidate gene on ChrII is SAMD4a known to be essential for correct tooth development in mice: mutants exhibit ectopic osteoblast differentiation and bone formation via Wnt-pathway upregulation (Li et al. 2011). This phenotype resembles the ectopic expression of Wnt3a in stickleback, which upregulates EDA and leads to the development of extra lateral plates (O'brown et al. 2015). On ChrIII, the SNP showing the strongest differentiation is near the DSPa gene. In humans, DSPa is associated with Skin fragility-woolly hair syndrome (Whittock et al. 2002), including skin and hair phenotypes similar to those caused by EDA mutations (Cui and Schlessinger 2006; Mikkola 2009). Moreover, DSPa is expressed in the ectoderm during zebrafish development and interacts with the Wnt/β-catenin signaling pathway (Giuliodori et al. 2018) implicated in stickleback plate development (Indjeian et al. 2016).

Supplementary Literature

Adhikari K et al. 2016. A genome-wide association scan in admixed Latin Americans identifies loci influencing facial and scalp hair features. Nat. Commun. 7:10815. doi:10.1038/ncomms10815.

Berner D. 2019. Allele frequency difference AFD - an intuitive alternative to FST for quantifying genetic population differentiation. Genes (Basel). 10:308.

Berner D et al. 2019. De novo sequencing, assembly, and annotation of four threespine stickleback genomes based on microfluidic partitioned DNA libraries. Genes (Basel). 10:426.

Chen, Y et al. 2012. A SNX10/V-ATPase pathway regulates ciliogenesis in vitro and in vivo. Cell Res. 22:333–345. doi:10.1038/cr.2011.134.

Cui CY, Schlessinger D. 2006. EDA signaling and skin appendage development. Cell Cycle 5:2477–2483. doi:10.4161/cc.5.21.3403.

Ferretti L, Ramos-Onsins SE, Pérez-Enciso M. 2013. Population genomics from pool sequencing. Mol. Ecol. 22:5561–5576. doi: 10.1111/mec.12522.

Giuliodori A et al. 2018. Loss of cardiac Wnt/β-catenin signalling in desmoplakin-deficient AC8 zebrafish models is rescuable by genetic and pharmacological intervention. Cardiovasc. Res. 114:1082–1097. doi:10.1093/cvr/cvy057.

Glazer AM, Killingbeck EE, Mitros T, Rokhsar DS, Miller CT. 2015. Genome assembly improvement and mapping convergently evolved skeletal traits in sticklebacks with genotyping-by-sequencing. G3-Genes Genomes Genet. 5:1463–1472. doi: 10.1534/g3.115.017905.

Indjeian VB et al. 2016. Evolving new skeletal traits by cis-regulatory changes in bone morphogenetic proteins. Cell . 164:45–56. doi:10.1016/j.cell.2015.12.007.

Kim SK. 2018. Identification of 613 new loci associated with heel bone mineral density and a polygenic risk score for bone mineral density, osteoporosis and fracture. PLoS ONE. 13:1–20. doi:10.1371/journal.pone.0200785.

Li J et al. 2011. SMAD4-mediated WNT signaling controls the fate of cranial neural crest cells during tooth morphogenesis. Development. 138:1977–1989. doi:10.1242/dev.061341.

Mikkola ML. 2009. Molecular aspects of hypohidrotic ectodermal dysplasia. Am. J. Med. Genet. 149:2031–2036. doi:10.1002/ajmg.a.32855.

Mills MG, Greenwood AK, Peichel CL. 2014. Pleiotropic effects of a single gene on skeletal development and sensory system patterning in sticklebacks. Evodevo. 5:1–10. doi:5\r10.1186/2041-9139-5-5.

O'Brown NM, Summers BR, Jones FC, Brady SD, Kingsley DM. 2015. A recurrent regulatory change underlying altered expression and Wnt response of the stickleback armor plates gene EDA. Elife. 4:e05290. doi: 10.7554/eLife.05290.

Thisse B, Thisse C. 2004. Fast Release Clones: A High Throughput Expression Analysis (ZFIN Direct Data Submission). Retrieved 22 January 2020, from http://zfin.org

Whittock NV et al. 2002. Compound heterozygosity for non-sense and mis-sense mutations in desmoplakin underlies skin fragility/woolly hair syndrome. J. Invest. Dermatol. 118:232–238. doi:10.1046/j.0022-202x.2001.01664.x.
